# Surgical Outcomes following Reoperation for Recurrent Intracranial Meningiomas

**DOI:** 10.3390/jcm13123356

**Published:** 2024-06-07

**Authors:** Shunya Hanakita, Soichi Oya

**Affiliations:** Department of Neurosurgery, Saitama Medical Center, Saitama Medical University, Kawagoe 3500086, Japan; hanakita-s@umin.ac.jp

**Keywords:** recurrent meningioma, re-do surgery, surgery-related complications

## Abstract

**Background:** We sometimes encounter refractory meningioma cases that are difficult to control, even after achieving a high resection rate or following radiation therapy (RT). In such cases, additional surgical resection might be attempted, but reports regarding outcomes of re-do surgery for recurrent meningiomas are scarce. **Methods:** This study was a retrospective review of patients who underwent re-do surgery for recurrent meningiomas. The risks of re-doing surgery were statistically analyzed. A comparative analysis between the patients who underwent primary surgery for intracranial meningiomas was also performed. Twenty-six patients underwent re-do surgeries for recurrent meningiomas. **Results:** At first re-do surgery, gross total resection was achieved in 20 patients (77%). The disease-free survival rate after the first re-do surgery was calculated as 73/58/44% at 1, 2, and 5 years, respectively. A significant factor affecting longer disease-free survival was WHO Grade 1 diagnosis at first re-do surgery (*p* = 0.02). Surgery-related risks were observed in 10 patients presenting a significant risk factor for skull base location (*p* = 0.04). When comparing with the risk at primary surgery, the risks of surgical site infection (*p* = 0.04) and significant vessel injury (*p* < 0.01) were significantly higher for the re-do surgery. **Conclusions:** Re-do surgery could increase surgery-related risks compared to the primary surgery; however, it could remain a crucial option, while the indication should be carefully examined in each case.

## 1. Introduction

Intracranial meningiomas are the most common brain tumors, accounting for about 30–40% of all primary intracranial tumors [1,2]. The majority of meningiomas are benign, and approximately 80% of meningiomas are WHO Grade 1. Surgical resection aiming to achieve gross total resection (GTR) is the standard of care for intracranial meningiomas; however, tumors recur even after GTR [1]. Thus, postoperative radiation therapy (RT), including stereotactic radiosurgery (SRS) or conventional radiation therapy, provides an acceptable tumor control rate, especially for WHO Grade 1 (higher than 90% at 10 years) [1]. On the other hand, WHO Grade 2 and 3 meningiomas are known for refractory characters against multidisciplinary therapy [1,2,3]. In such cases, additional surgical resection must be considered, although re-do surgeries are challenging for neurosurgeons because of the risks of surgery-related complications that may arise. Although there are theoretical concerns regarding the risk and surgical outcomes, reports of the actual risks of repeat surgery for recurrent intracranial meningiomas are limited [3,4,5,6]. Therefore, this study retrospectively analyzed the surgical outcomes of patients who underwent re-do surgery for recurrent intracranial meningiomas to clarify the characteristics of surgery-related risks and identify potential dangers that we should carefully consider before deciding on re-do surgery.

## 2. Materials and Methods

### 2.1. Study Population

We retrospectively reviewed our institutional database for 262 patients with 267 meningiomas who underwent surgery for intracranial recurrent meningiomas between January 2011 and December 2023. Follow-up was included up to April 2024. Cases were restricted to those performed by the senior author (S.O.). Four patients who were treated with intentionally staged procedures and had a history of more than 2 surgeries at other hospitals or had multiple meningiomas were excluded from this study. In addition, 4 patients who received endoscopic endonasal tumor resection were also excluded. Among 254 patients, 26 patients were included in this study. To clarify whether re-do surgery affected the patient’s outcomes, a comparative analysis was conducted between the primary resection and the re-do surgery. The methods for our retrospective study were approved by the Institutional Review Board (IRB number 2023-41).

### 2.2. Comparison Cohort

A comparative cohort of patients was generated from our database to compare surgical outcomes between primary and re-do surgeries. The patients that were included in the analysis had the following variables recorded: tumor diameter, location of the tumor, extension of the surgical resection, symptomatic radiographic changes on immediate postop MR imaging, and surgery-related complications, including neurological deterioration, postoperative hemorrhage, surgical site infection, CSF leakage, wound dehiscence, and frequency of seizure.

### 2.3. Evaluation of Patient Outcomes and Statistical Analysis

After surgery, follow-up clinical examinations were performed at our hospital with serial MR imaging within a 6- or 12-month interval. Tumor control was defined as the absence of tumor enlargement ≥2 mm in any direction or the progression of recognizable masses in new locations. The disease-free survival (DFS) rate was calculated using the Kaplan–Meier method. A log-rank test was used for univariate analysis to evaluate factors that could affect DFS. For factors related to surgical complications, including perioperatively delayed onset events (such as wound dehiscence and CSF leakage), tumor WHO Grade, modified Rankin scale at discharge and six months postoperatively, and extension of surgical resection, the chi-square test and Fisher’s exact tests were performed. A *p*-value < 0.05 was considered significant. All analyses were performed using JMP Pro, version 16 software (SAS Institute Inc., Cary, NC, USA).

## 3. Results

### 3.1. Study Population and Characteristics of Patients

A total of 254 patients with intracranial meningiomas were operated on during this period, of which 26 patients (10%) received at least one re-do operation for recurrent meningioma. Six patients (23%) had their primary surgery at another hospital and were referred to our department for re-do surgery due to tumor recurrence. The baseline characteristics of the 26 patients are summarized in Table 1. The presenting symptoms in the patients before primary surgery varied and included visual disturbance in seven (27%), cognitive dysfunction in six (23%), seizure in five (22%), and motor weakness in six (23%). The mean age at primary operation was 59 years old (SD = 12.7; range: 34–76), and at re-do surgery, the mean age was 64.7 years old (SD = 10.6; range: 44–79). The mean follow-up period after primary resection, including at affiliated hospitals, was 77.1 months (SD = 45.2; range: 6–192). The mean interval between the primary and re-do surgery was 63.7 months (SD = 55.9; range: 5–240). Among these patients, 11 (42%) had a history of radiation therapy for salvage treatment for residual tumors or tumor recurrence after initial resection: 3 had intensity-modulated radiotherapy (IMRT), 6 had stereotactic radiotherapy (SRT), 1 had SRS, and 1 had IMRT and SRS. The mean interval between the primary radiation therapy and re-do surgery was 54.8 months (SD= 41.6; range: 12–120).

The tumor characteristics are presented in Table 2. The median largest tumor diameter at the first re-do surgery was 32 mm (SD = 13.4; range: 9–73). Magnetic resonance imaging (MRI) showed an increased T2 high intensity in 5 patients (19%), and peritumoral edema was detected in 16 patients (62%). The tumor location was at the skull base region in 13 patients and the non-skull base region in the other 13 patients. At the primary resection, the number of patients who had WHO Grade 1 and 2 tumors was 17 and 9, respectively. At the last follow-up, 4 (24%) of the 17 patients initially diagnosed with WHO Grade 1 meningiomas had a change in their pathological diagnosis to WHO Grade 2 meningiomas. During the primary surgery, GTR or near total resection (NTR) was achieved in 16 patients (62%), and subtotal resection (STR) was achieved in 10 patients (38%).

### 3.2. Surgical Outcome after Re-Do Surgery

The clinical outcomes after re-do surgery are summarized in Table 3.

**Figure 1:**
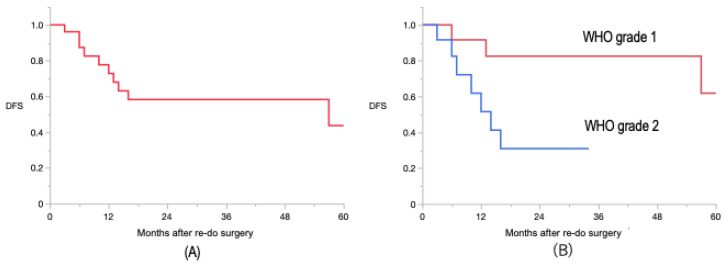
Kaplan–Meier curves of disease-free survival rate after the first re-do surgery. (**A**) The actuarial DFS rates were 73%, 58%, and 44% at 1, 2, and 5 years, respectively. (**B**) Kaplan–Meier curves of DFS stratified by WHO grading at the first re-do surgery showing significantly longer DFS for WHO Grade 1 patients (92%, 83%, and 62% at 1, 2, and 5 years, respectively).

A GTR or NTR was achieved in 20 patients (77%), and an STR was achieved in 6 patients (23%). At the last follow-up, 4 (24%) of the 17 initially diagnosed WHO Grade 1 meningioma patients had their pathological diagnosis changed to WHO Grade 2 meningiomas. No one was newly diagnosed with WHO Grade 3 meningiomas during this follow-up period. Among the 26 patients, 8 received adjuvant radiation therapy (6 by IMRT and 2 by SRS) following the first re-do surgery because their pathology was either newly diagnosed as WHO Grade 2 meningioma or their Ki-67 label index seemed to be aggressive (>10%). During follow-up, 18 patients did not show signs of further tumor recurrence after re-do surgery (among these 18, 5 underwent adjuvant radiation therapy following the re-do surgery); however, 8 patients required further treatment. Four patients underwent additional SRS, but three of them failed. Five patients were re-operated two or more times (three patients had a second re-do surgery, one patient had a third re-do surgery, and another patient had a fourth re-do surgery). The mean time between the first and second re-do surgery was 36 months (SD = 22.5; range: 15–72). When calculated using the Kaplan–Meier method, the DFS rates after the re-do surgeries were 73%, 58%, and 44% at 1, 2, and 5 years, respectively (Figure 1A). The factors associated with the DFS rate are presented in Table 4.

The only favorable factor for survival (*p* = 0.02) during the re-do surgery was the presence of a WHO Grade 1 meningioma. There was no significant difference in survival associated with the extension of resection at re-do surgery (GTR/NTR vs. STR, *p* = 0.36), history of radiation therapy before re-do surgery (*p* = 0.29), or location of the meningioma (skull base vs. non-skull base, *p* = 0.51). The DFS rates for WHO Grade 1 patients after the first re-do were 92%, 83%, and 62% at 1, 2, and 5 years, respectively (Figure 1B). In addition, a Ki-67 label index of less than 5% was a significant factor in achieving a longer DFS (*p* = 0.02).

**Figure 2:**
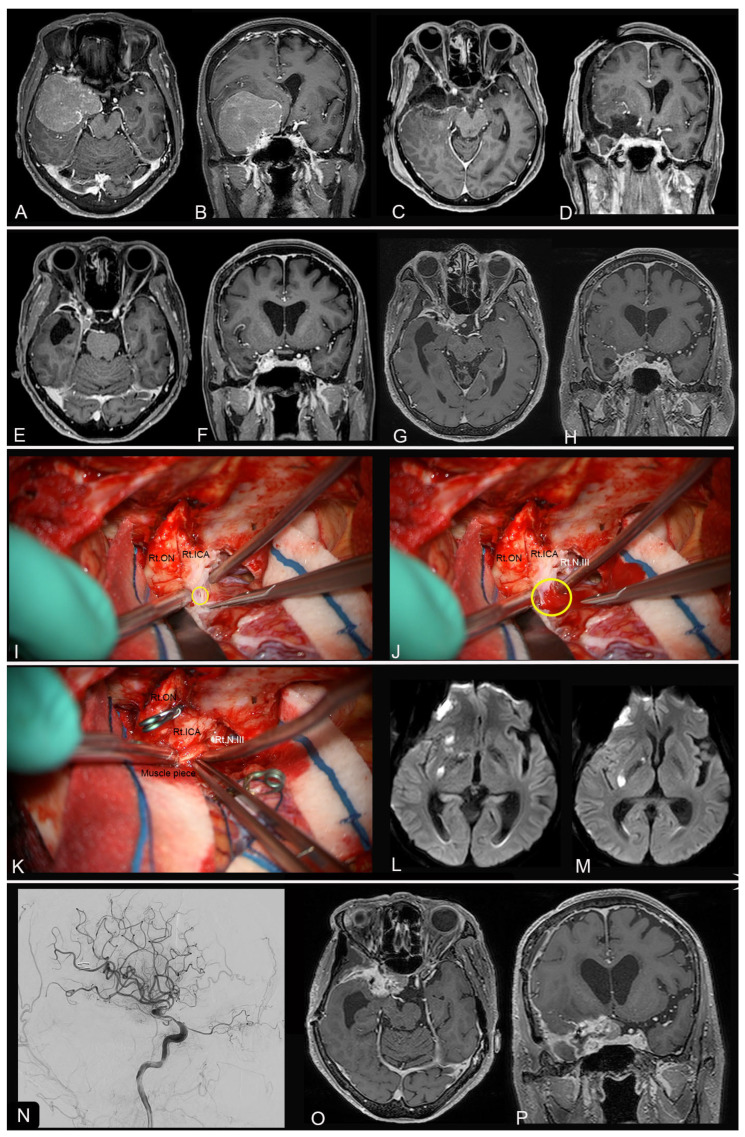
An illustrative case of surgical-related complication (ICA injury). (**A**,**B**) A woman in her 70s presented with visual disturbance and a right middle base meningioma (T1 Gd-enhanced MRI). (**C**,**D**) Primary surgery resulted in GTR without any neurological deterioration. (**E**,**F**) A small recurrence was detected 8 months after primary surgery, and stereotactic radiosurgery (SRS) was performed. (**G**,**H**) Further recurrence was detected during follow-up. A re-do surgery was attempted 20 months after the primary surgery. (**I**–**K**) Intraoperative view. (**I**) Small laceration (yellow circle) on the right ICA occurred due to severe adhesion. (**G**) Bleeding from the ICA started (yellow circle) but was controlled by double suction technique. (**K**) A direct suture and compression with a muscle piece could achieve hemostasis. (**L**,**M**) DWI on immediate postoperative MRI showed ischemic changes in the right basal ganglia region. (**N**) Postoperative internal angiography showed stenosis of the ICA (lateral view). The patient presented with transient motor weakness and diplopia, which completely resolved during follow-up. (**O**,**P**) The patient underwent additional SRS for the residual lesion.

As for surgery-related complications after re-do surgery, nine patients had neurological deterioration. Four patients developed new symptoms: one developed hemiparesis with facial palsy, one developed hemiparesis with oculomotor palsy, one developed aphasia, and one developed a seizure. The other five patients showed worsening existing symptoms: three exhibited oculomotor palsy, and two showed transient motor weakness. Consequently, five patients (19.2%) showed neurological deterioration after the first re-do surgery. In addition, one patient developed a wound infection, two patients developed CSF leakage (requiring surgery in one), and two patients developed a major vessel injury (internal carotid artery in one and superior straight sinus in one) (Figure 2). Radiographic changes (ischemia, hemorrhage, or contusion) on immediate postop MR imaging were found in 19 patients (73%), of which 10 were symptomatic (38%). There was no surgery-related mortality during the postoperative course. The location of the skull base was a significant risk factor for neurological deterioration following re-do surgery (*p* = 0.03) (Table 5).

### 3.3. Cohort Comparisons: Risk at Primary Surgery versus Re-Do Surgery

For this comparative study, two cohorts were made. The previously mentioned 26 patients who underwent re-do surgery were named Group A. Among this group, 6 patients were initially treated at other hospitals, and the other 20 patients who underwent primary surgery at our hospital were collected and named as Group B to analyze their risk at primary surgery. In addition to these 20 patients who received their primary resection at our hospital, 228 patients who received only one surgical resection were selected and included as part of Group B (*n* = 248). The comparison between the groups is summarized in Table 6.

As for surgical-related complications, the frequency of surgical infections (7.7% vs. 1.2%, *p* = 0.02), injury of major vessels (7.7% vs. and 0.4%, *p* < 0.01), and symptomatic radiographic imaging on postoperative MRIs (38% vs. 19%, *p* = 0.03) were significantly higher in Group A. There was no significant difference between groups in terms of other variables, including CSF leakage, hydrocephalus requiring a shunt, symptomatic venous infarction, postoperative hematoma requiring evacuation, or other medical complications (Table 7).

## 4. Discussion

This study aimed to analyze the surgical outcomes of resection of recurrent intracranial meningioma and identify predictive factors for achieving longer DFS and risk factors for surgery-related complications at re-do surgeries. First, we found that the DFS rate was influenced by its WHO grading and Ki-67 label index. It is widely known that a WHO Grade 2 meningioma is highly resistant to treatment, regardless of the extent of GTR achieved at primary resection or adjuvant IMRT [1,7,8,9,10]. Even for WHO Grade 1 patients, a Ki-67 index > 5% is a risk factor, as previously reported [11,12,13]. Second, the incidence of surgical site infection and significant vessel injury showed a substantial increase compared to the results of the primary surgery performed by the same senior author within the same time frame.

The primary treatment strategy for intracranial meningioma is surgical resection that aims for GTR; however, there is a potential risk of postoperative neurological deterioration when attempting to increase the extent of the resection [1,2]. In addition, even for benign meningiomas, GTR does not mean a permanent cure. The local recurrence rate ranges from 20 to 39% at 10 years and 24 to 60% at 15 years [1]. Therefore, RT has been widely accepted as one of the effective treatment options for intracranial meningiomas [1,2]. During the last two decades, the efficacy and safety of radiation therapy, especially SRS, have been consistently reported [14]. Radiation therapy provides favorable outcomes for benign intracranial meningioma, especially SRS; however, there are certain patients who suffer from tumor recurrence even after GTR and RT [15].

Repeat RT for recurrent meningiomas should be approached with caution in such cases because of the adverse effect of reirradiation. A few studies have reported excellent tumor control via this method while maintaining acceptable levels of adverse events [16,17]. Then, re-do surgery could be considered an alternative salvage treatment. However, from a surgical aspect, re-do surgery often carries significant risks due to the presence of adhesions surrounding the previous tumor cavity and a high risk of wound dehiscence and surgical site infection. Compared to the well-reported primary surgical results of intracranial meningiomas, reports of the surgical results of re-do surgery are scarce [3,4,5,18]. We have summarized past studies concerning the surgical results of repeat surgery for recurrent intracranial meningioma in Table 8.

According to previous reports, the neurological permanent complication rates ranged from 12.8% to 19.6%, similar to our results. It has been reported that there is a risk of CSF leakage or pseudomeningocele occurring at a rate of 3–12.5%, with a specific rate of 7.7% in our study. Additionally, wound dehiscence or wound infection occurs at a rate of 4.5–10.2%, comparable to our results (7.7%). Therefore, the timing of re-do surgeries remains a matter of debate, especially when the patients have no clinical symptoms. On the other hand, especially for Grade 2 or 3 meningiomas, high rates of recurrence are known, and reoperation and a combination of radiation therapy could not overcome these refractory pathologies [1,2,7,8,9,10,19,20]. Despite the high risks of complications, there is a certain situation in which surgical resection plays a role as a last resort for tumor recurrence. In our series, among the WHO Grade 1 patients, the Ki-67 label index <5% was a predictive factor for longer tumor control, as previously reported [11,12,13]. Meningioma could recur even after observation over 10 years, so a longer follow-up of decades is desirable.

Limitations: This study has several limitations. First, this is a retrospective study in a single institute. Second, some patients had a primary resection at other hospitals, so the surgery-related risk following primary surgery could be underestimated due to suboptimal data collection.

## 5. Conclusions

Re-do surgeries for intracranial recurrent meningioma can play an important role in managing refractory recurrence meningioma, but there are increasing risks of surgery-related complications. The timing of intervention should be carefully examined in each case.

## Figures and Tables

**Table 1:** Patient characteristics.

Variable	Value
No. of patients	26
Male/female	11/15
Mean follow-up period (months; range)	77.1, 6–192
Mean age at primary surgery (yrs; range)	59, 34–76
Mean age at first re-do surgery	64.7, 44–79
Mean interval between primary surgery and first re-do surgery (months; range)	63.7, 5–240
No. of patients who underwent RTx before first re-do surgery	11 (41%)
Mean interval between primary surgery and RTx (months; range)	56.7 (12–120)
Mean interval between primary RTx and first re-do surgery (months; range)	54.8 (12–120)
RTx performed before first re-do surgery	
IMRT	3
SRT/SRS	7
Initial symptoms, *n* (%)	7 (27%)
Visual disturbance	6 (23%)
Cognitive disfunction	6 (23%)
Seizure	5 (19%)
Motor weakness	1 (3.8%)
Aphasia	1 (3.8%)
Exophthalmos	5 (19%)
Incidental finding	5 (19%)
Extension of resection at primary surgery	
GTR/NTR	16 (62%)
STR	10 (38%)

GTR: gross total resection, IMRT: intensity-modulated radiotherapy, NTR: near total resection, RTx: radiation therapy, SRS: stereotactic radiosurgery, SRT: stereotactic radiotherapy, STR: subtotal resection.

**Table 2:** Tumor characteristics.

Variable	Value
Largest diameter at first re-do surgery (mm; range)	32 (9–73)
Peritumoral edema	16 (62%)
MRI T2 high signal intensity	5 (19%)
Tumor location	
Skull base region	13
Anterior skull base region	
Spheno-orbital	1
Sphenoid ridge	6
Middle skull base region	
Middle base	3
Posterior skull base region	
Posterior clinoid	1
Petrotentorial	1
Petroclival	1
Non-skull base region	13
Parasagittal sinus	7
Tentorium	2
Convexity	2
Falx	1
Ventricle	1
Pathological diagnosis at primary surgery	
WHO Grade 1	17 (65%)
Grade 2	9 (35%)

**Table 3:** Surgical outcomes of the re-do surgery.

Variable	Value
Extension of resection at primary surgery	
GTR/NTR	20 (77%)
STR	6 (23%)
No. of patients who underwent additional treatment	11 (42%)
Additional RTx after first re-do surgery	10
SRT	6
IMRT	4
No. of patients who received more than 2 re-do surgeries	5
No. of patients who had a second re-do surgery	3
No. of patients who had a third re-do surgery	1
No. of patients who had a fourth re-do surgery	1
Mean interval between the first and second re-do surgery (months; range)	36 (15–72)
Surgery-related complication	
Newly developed symptoms	4
Hemiparesis and facial palsy	1
Hemiparesis and oculomotor palsy	1
Aphasia	1
Seizure	1
Worsening of existing symptoms	6
Oculomotor palsy	1
Hemiparesis	3 (2 transient)
Hemianopia	2
Radiographical changes on postop MRI	19 (73%)
Symptomatic radiographical changes	10 (38%)
Major vessel injury	2
ICA	1
SSS	1
Wound dehiscence	1
CSF leakage	2

ICA: internal carotid artery, RTx: radiation therapy, SSS: superior sagittal sinus. Surgery-related complications include surgical site infection, CSF leakage, and major vessel injury.

**Table 4:** Univariate analysis of factors affecting disease-free survival rate after the first re-do surgery.

Variable	*p*-Value
Patient characteristic	
Male/female	0.93
Age > 60	0.58
History of RTx before first re-do surgery	0.29
Extension of resection GTR/NTR vs. STR	0.36
Tumor characteristic	
Skull base location	0.51
Max tumor diameter >3 cm at first re-do surgery	0.77
WHO Grade 1 meningioma at first re-do surgery	0.02 *
MRI T2 high signal intensity	0.78
Peritumoral edema on MRI	0.30

* means the *p*-value is significant.

**Table 5:** Univariate analysis of risk factors affecting surgical complications.

	Any Complication	Neurological Deterioration	Surgery-Related Complication (Wound Dehiscence, CSF Leak, Major Vessel Injury, etc.)
Variable	*p*-Value	*p*-Value	*p*-Value
Patient characteristic			
Male/female	0.95	0.85	0.97
Age > 60	0.28	0.12	0.38
Any symptom at re-do surgery	0.43	0.42	0.66
History of RTx before re-do surgery	0.052	0.26	0.12
Extension of resection GTR/NTR vs. STR	0.25	0.51	0.69
Tumor characteristic			
Skull base location	0.04 *	0.03 *	0.83
Max tumor diameter >3 cm at first re-do surgery	0.02 *	0.19	0.69
WHO Grade 1 meningioma at first re-do surgery	0.95	0.32	0.35
MRI T2 high signal intensity	0.19	0.35	0.69
Peritumoral edema on MRI	0.19	0.13	0.53

* means the *p*-value is significant.

**Table 6:** Comparison of patients and tumor characteristics in Groups A and B.

Variable	Group A (Re-Do op)	Group B (Primary op)
Patient characteristics		
No. of patients	26	248
Male/female	11/15	71/171
Mean age at surgery (yrs; range)	64.7, 44–79	62.0, 26–85
Extension of resection		
GTR/NTR	16 (62%)	202 (82%)
STR	10 (38%)	46 (19%)
Tumor characteristics		
Largest diameter at surgery (mm; range)	32 (9–73)	39 (6–88)
Peritumoral edema on MRI	16 (62%)	142 (57%)
MRI T2 high signal intensity	5 (19%)	109 (41%)
Tumor location		
Skull base region	13 (50%)	123 (50%)
Non-skull base region	13 (50%)	125 (49%)
Pathological diagnosis		
WHO Grade 1	17 (65%)	218 (88%)
Grade 2	9 (35%)	30 (12%)

**Table 7:** Comparison of surgery-related risks between the re-do surgery (Group A) and the primary surgery (Group B).

Variable	Group A (Re-Do op)	Group B (Primary op)	*p*-Value
Number of patients	26	248	
Neurological deterioration (including transient symptoms)	9 (35%)	65 (26%)	0.36
CSF leak requiring a lumbar or repair procedure	2 (7.7%)	6 (2.4%)	0.13
Hydrocephalus requiring a shunt procedure	0	2 (0.8%)	0.65
Surgical site infection/wound dehiscence	2 (7.7%)	3 (1.2%)	0.02 *
Symptomatic trouble of venous drainage system	0	3 (1.2%)	0.57
Injury of major vessels	2 (7.7%)	1 (0.4%)	<0.01 *
Symptomatic contusion/ischemic changing on MRI	10 (38%)	49 (20%)	0.03 *
Newly developed seizure (requiring antiseizure medication)	1 (3.9%)	5 (2.3%)	0.63
Postop hematoma requiring evacuation	0	1 (0.4%)	0.75
Medical complication: symptomatic PE	0	2 (0.8%)	0.65

* means the *p*-value is significant.

**Table 8:** Previous reports of re-do surgery for recurrent intracranial meningioma.

Author/Year	No. of Pts	Tumor Location: Non-Skull Base /Skull Base	WHO Grade 1/2/3 (%)	RT Before the First Re-Do Surgery	Surgical Complications after the Re-Do Surgery (%)		Risk Factors
Magill et al., 2019 [4]	67	Non-skull base	22/51/27	24 (36%)	Hydrocephalus Hemorrhage CSF leakage/pseudo meningocele Wound dehiscence/infection Neurological deterioration	1.5% 1.5% 3% 4.5% 19.4%	The middle third of the sagittal plane
Magill et al., 2019 [5]	78	Skull base	72/22/6	16 (21%)	Hydrocephalus Hemorrhage CSF leakage/pseudo meningocele Wound dehiscence/infection Neurological deterioration	10% 3.8% 7.7% 10.2% 12.8%	Posterior fossa
Lemée et al., 2020 [3]	114	N.A.	68/19/13	N.A.	Hemorrhage Infection Neurological deterioration	2.7% 3.5% 16.6%	N.A.
Richardson et al., 2021 [18]	56	61%/39%	48/45/5	N.A.	Hemorrhage CSF leakage/pseudo meningocele Infection Neurological deterioration	1.8% 12.5% 5.4% 19.6%	N.A.
Present study	26	50%/50%	65/35/0	10 (38%)	Hydrocephalus Hemorrhage CSF leakage/pseudo meningocele Wound dehiscence/infection Neurological deterioration	0% 0% 7.7% 7.7% 19%	Skull base location

N.A. nothing available.

## Data Availability

The data presented in this study are available upon request from the corresponding author. The data are not publicly available due to data privacy.
